# The Pivotal Potentials of Scorpion *Buthus Martensii Karsch-*Analgesic-Antitumor Peptide in Pain Management and Cancer

**DOI:** 10.1155/2020/4234273

**Published:** 2020-10-29

**Authors:** Seidu A. Richard, Sylvanus Kampo, Marian Sackey, Maite Esquijarosa Hechavarria, Alexis D. B. Buunaaim

**Affiliations:** ^1^Department of Medicine, Princefield University, P.O. Box MA128, Ho, Ghana; ^2^Department of Anesthesia and Critical Care, School of Medicine, University of Health and Allied Sciences, Ho, Ghana; ^3^Department of Pharmacy, Ho Teaching Hospital, P.O. Box MA-374, Ho, Ghana; ^4^Department of Surgery, School of Medicine and Health Science, University for Development Studies, Tamale, Ghana

## Abstract

Scorpion *Buthus martensii Karsch* -analgesic-antitumor peptide (BmK AGAP) has been used to treat diseases like tetanus, tuberculosis, apoplexy, epilepsy, spasm, migraine headaches, rheumatic pain, and cancer in China. AGAP is a distinctive long-chain scorpion toxin with a molecular mass of 7142 Da and composed of 66 amino acids cross-linked by four disulfide bridges. Voltage-gated sodium channels (VGSCs) are present in excitable membranes and partakes in essential roles in action potentials generation as compared to the significant function of voltage-gated calcium channels (VGCCs). A total of nine genes (Na_v_1.1–Na_v_1.9) have been recognized to encode practical sodium channel isoforms. Na_v_1.3, Na_v_1.7, Na_v_1.8, and Na_v_1.9 have been recognized as potential targets for analgesics. Na_v_1.8 and Na_v_1.9 are associated with nociception initiated by inflammation signals in the neuronal pain pathway, while Na_v_1.8 is fundamental for neuropathic pain at low temperatures. AGAP has a sturdy inhibitory influence on both viscera and soma pain. AGAP potentiates the effects of MAPK inhibitors on neuropathic as well as inflammation-associated pain. AGAP downregulates the secretion of phosphorylated p38, phosphorylated JNK, and phosphorylated ERK 1/2 *in vitro*. AGAP has an analgesic activity which may be an effective therapeutic agent for pain management because of its downregulation of PTX3 via NF-*κ*B and Wnt/beta-catenin signaling pathway. In cancers like colon cancer, breast cancer, lymphoma, and glioma, rAGAP was capable of blocking the proliferation. Thus, AGAP is a promising therapy for these tumors. Nevertheless, research is needed with other tumors.

## 1. Introduction

Scorpion *Buthus martensii Karsch* (BmK) constitutes an integral portion of Chinese traditional medicine for the treatment of several diseases like tetanus, tuberculosis, apoplexy, epilepsy, spasm, migraine headaches, rheumatic pain, and cancer [1, 2]. Scorpions venom composes of a complex combination of low molecular weight bioactive molecules as well as small peptides and enzymes [1–4]. Several distinctive toxic peptides extracted from scorpion venom have diverse functions [1, 3, 4]. Analgesic-antitumor peptide (AGAP) was extracted from the venom of Scorpion BmK approximately 20 years ago [1, 3, 4]. Studies on the expression as well as purification of AGAP were conducted using *Escherichia coli* (*E. coli*), while the biological action of AGAP was experimented in mice [5].

Recombinant AGAP (rAGAP) is made up of small ubiquitin-related modifier-AGAP (SUMO-AGAP) which is associated with a hexahistidine tag by *E. coli* [6]. Thus, rAGAP is a fusion protein comprising a hexahistidine (His6) tag, SUMO, and AGAP. Also, rAGAP was oversecreted in *E. coli* [3]. Studies demonstrated that AGAP may be a Na^+^-channel specific inhibitor because it was capable of inhibiting mRNA transcription of voltage-gated sodium channels (Na_v_) [6, 7]. Electrophysiological studies using hNa_v_1.4, hNa_v_1.5, hNa_v_1.7, and hNa_v_1.8 revealed that AGAP is possibly a *β*-type scorpion toxin rather than an *α*-type toxin [8].

AGAP lengthens the survival of mice with engrafted *Ehrlich ascites* (*E. ascites*) tumor cells significantly and also subdued the growth of S-180 fibrosarcoma effectively [4]. In comparison with cyclophosphamide, it was observed that AGAP had more affinity for tumor cells and less harm for healthy cells [4]. Studies have demonstrated that AGAP has analgesic and antitumor potentials [1–4, 6]. Although scorpion toxin contains numerous toxic polypeptides with dissimilar functions as reviewed by Wang et al. [9], this review explicitly focuses on AGAP. We elucidate the cardinal analgesic and antitumor potentials of AGAP with a focus on the key signaling mechanisms via which it functions. Most of the articles reviewed were indexed in PubMed and PubMed Central with strict inclusion criteria being analgesic and antitumor potentials of BmK AGAP. The “Boolean logic” was utilized to search for articles on the subject matter. The key search words were analgesia and/or AGAP, cancer/tumor and/or AGAP, anticancer/antitumor and/or AGAP as well as AGAP signaling pathways. None peer-reviewed articles and news files were excluded.

## 2. Structure and Functions of BmK AGAP

AGAP is a distinctive long-chain scorpion toxin with a molecular mass of 7142 Da. It composes of 66 amino acids cross-linked by four disulfide bridges such as Cys12–Cys63, Cys16–Cys36, Cys22–Cys46, and Cys26-Cys48 [10]. This structure suggests that AGAP is a suitable model to identify an analgesic domain. AGAP demonstrated soluble secretions in *E. coli* after it was refined and duplicated. Subsequently, its bioactivity was investigated in an animal experiment [4, 11]. Nevertheless, the structure-functional association between Na_v_s and AGAP, leading to the analgesic effects, still needs further studies. A total of four disulfide bridges in AGAP existed after analysis with site-directed mutagenesis in an *E. coli* model [10]. The four disulfide bonds in the AGAP were modified using 12 mutants, comprising 8 single mutants and 4 double mutants, in which Ser was substituted for Cys [10]. Also, disulfide bonds are the only common covalent cross-links in polypeptide chains [10].

The polypeptide chain in AGAP can assume numerous conformations and the sequence of its amino acid residues targets folding in a specific conformation [10]. The fundamental components associated with folding were the formation of disulfide bonds which restricts the quantity of folded conformations of a polypeptide chain [10, 12]. Furthermore, the principal consequence of disulfide bonds was stabilizing the protein foisted distance as well as angle restrictions between the C^*β*^ and S^*γ*^ atoms of the joined cysteine residues [10, 13]. The protein structure, conformational stability, catalytic activity, and folding are thus useful for further studies into AGAP [10, 14]. Also, mutants in the disulfide bridges regulating the analgesic action are visible on the molecule exterior in two domains (core and NC domains). The Core domain comprises Gly-17, Arg-18, Trp-38, and Asn-44. The Gly-17 and Arg-18 are in the long-loop linking the secondary structure motifs of the molecule *α*/*β*-core. On the other hand, NC-domain comprises five-residue-turn (residues 8–12) as well as the C-terminal region (residues 56–64) [10, 15].

## 3. BmK AGAP and Voltage-Gated Channels

Voltage-gated sodium channels (VGSCs) facilitate the internal sodium current in the brain and peripheral nerves [16]. VGSCs are thus crucial in triggering as well as propagation of action potentials in excitable tissues such as the brain and peripheral nerves [17]. AGAP is an ion channel regulator with diverse activities on a variation of neuronal ion channels such as high-voltage-activated (HVA), low-voltage-activated (LVA) calcium channels as well as tetrodotoxin-resistant (TTX-R) sodium channels [16]. A total of nine genes (Na_v_1.1–Na_v_1.9) have been recognized to encode practical sodium channel isoforms [8]. Na_v_1.4 encoded by SCN4A was primarily secreted by skeletal muscle. It was necessary for the initiation as well as propagation of the action potential necessary for skeletal muscle contraction. On the other hand, Na_v_1.3, Na_v_1.7, Na_v_1.8, and Na_v_1.9 have been recognized as potential targets for analgesics [8].

Studies have demonstrated that peripheral nerve injury often aggravates chronic pain which was associated with hyperexcitability of sensory neurons in dorsal root ganglia (DRG) [16, 18–20]. Caffrey et al. identified two types of sodium currents in small-diameter neurons of rat DRG [21]. Furthermore, studies have shown that Na_v_1.8 and Na_v_1.9 are composed of the basic pattern of TTX-R sodium channel subtypes that are extremely secreted in peripheral sensory neurons [16, 18, 22]. They are both believed to partake in significant functions during ectopic expression via neuronal bodies as well as axons subsequent to peripheral nerve injury [22]. These characteristics perhaps make them possible molecular targets for analgesic drugs [16, 23]. Studies have further shown that both TTX-R subtypes, Na_v_1.8 and Na_v_1.9, are associated with nociception initiated by inflammation signals in neuronal pain pathway while Na_v_1.8 was fundamental for neuropathic pain at low temperatures [24, 25].

Li et al. confirmed that AGAP might attenuate pain by blocking TTX-R channels in small-diameter DRG neurons [16]. They demonstrated that 1000 nM AGAP decreased the Nav1.8 as well as Na_v_1.9 currents while the inhibitory proportion of Na_v_1.8 as well as Na_v_1.9 currents were 59.4 ± 5.1 and 33.7 ± 6.6%, correspondingly. They further suggested that the consequence of AGAP on Na_v_1.8 channels was greater than that of Na_v_1.9 channels [16]. Jarvis et al. indicated that Na_v_1.8 was essential in the development and/or continuation of nerve injury-induced pain [26]. They specified that numerous Na_v_1.8-specific blockers are associated with analgesic activities in neuropathic pain [26]. Li et al. established that Na_v_1.8 might be a potential target of AGAP in inflammatory as well as neuropathic antinociceptive targets [16]. Genetic and functional studies have demonstrated that Na_v_1.7 was predominantly involved in pain signaling in humans [27]. Studies further showed that Na_v_1.7 was linked with erythromelalgia, paroxysmal life-threatening pain disorder, and congenital insensitivity to pain [27, 28]. Xu et al. demonstrated that AGAP potently blocked the action of both hNa_v_1.7 and hNa_v_1.8, signifying that both channel isoforms were involved in the analgesic mechanisms of AGAP [8].

Furthermore, studies have shown that Na_v_1.9 channels are crucial intermediators of inflammation than neuropathic pain and participation in hyperexcitability of nociceptors during inflammatory pain [16, 29–31]. Also, hyperexcitability occurred during the silencing of Na_v_1.9 resulting in the loss of depolarizing subsequent to hyperpolarizing shift in resting potential and eradicated resting inactivation on TTX-S NaCl channels [32]. Xu et al. demonstrated that AGAP showed a notable blockade influence on both hNa_v_1.4 and hNa_v_1.5, which accounts for its toxicity in skeletal and cardiac muscles [8]. They further proved that W38G mutation drastically reduced the blockade of I_Nap_ with an augmented calculated IC_50_ value of 60,000-fold for hNa_v_1.4 and 420-fold for hNa_v_1.5, respectively. They again indicated that the decreased blockade of W38G on hNa_v_1.4 and hNa_v_1.5 appeared as a result of its decreased inhibitory activities on both channels than its alteration on kinetic activities. Nevertheless, they explained that Trp^38^ could be the fundamental amino acid that aids in AGAP communication with hNa_v_1.4 and hNa_v_1.5 [8].

Liu et al. indicated that AGAP potently blocked voltage-gated calcium channels (VGCCs), particularly high-voltage triggered calcium currents in rat DRG neurons [33]. Studies have shown that AGAP reduced HVA calcium channels, specifically N-type calcium currents, while N-type calcium channels are secreted at the presynaptic level and may have more effects on EPSC/EPSP than other calcium channel types [16, 34]. Nevertheless, voltage-gated L-type calcium channels are capable of sustaining longer-lasting depolarizations leading to decreased firing threshold, control repetitive firing, and influencing regenerative action potentials [16]. Li et al. indicated that antinociceptive consequence of AGAP might be due to its precise alteration of voltage-gated ion channels of sensory neurons [16]. Payandeh et al. indicated that VGSCs are present in excitable membranes and partakes in essential roles in action potentials generation as compared to the significant function of VGCCs [17].

## 4. Nerve Conduction and the Role of AGAP in Pain

Studies have proven that nerve injury can trigger painful hyperalgesia subsequent to allodynia [1, 35, 36]. Also, sensitization of the peripheral nociceptor triggered by inflammation or injury manifests as hyperalgesia [1]. Hyperalgesia is an exaggerated pain reaction to a noxious stimulus, while allodynia is the perception of a nonnoxious stimulus as noxious [1, 37]. Furthermore, peripheral sensitization facilitates the firing of small-diameter sensory neurons that communicate information regarding noxious stimuli to the dorsal horn of the spinal cord and augments synaptic activities [36, 38]. This in turn triggers central sensitization, a major cellular mechanism resulting in the transformation of acute nociceptive injuries into chronic pain [1].

Central sensitization is depicted with upsurges in the excitability of neurons and augmentation of reactions to nociceptive or/and nonnociceptive stimuli [1, 37]. Central sensitization plays a crucial role in the pathogenesis of chronic pain [39]. The initiation and continuance of central sensitization are determined by maladaptive modifications in the secretion, distribution, and action of ion channels, receptors, and intracellular signal transduction pathways [1, 39]. Central sensitization is also one of the principal triggers of behavior hyperalgesia under pathologic conditions [37, 39]. Liu et al. indicated that AGAP had a sturdy inhibitory influence on both viscera and soma pain [4].

**Figure 1:**
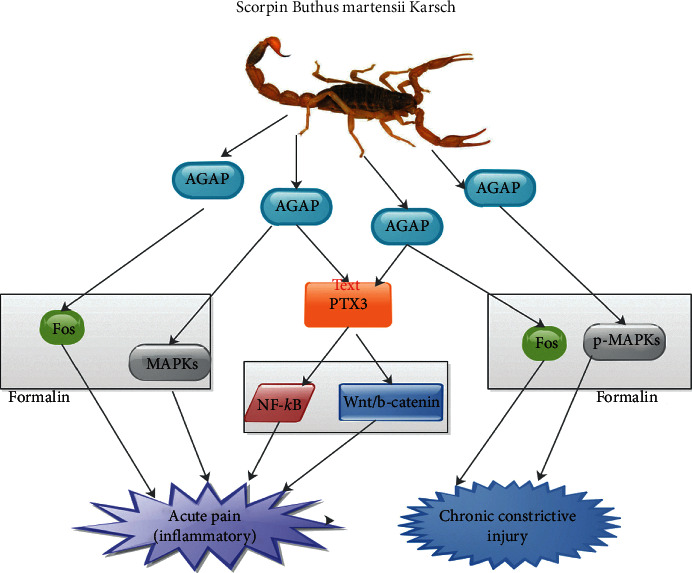
An illustration showing the inhibitory pathways via which AGAP elicited analgesia. AGAP is capable of resolving acute inflammatory pain and chronic constrictive injury via MAPS and Fos pathways in formalin.

Mao et al. demonstrated that preintraplantar administration AGAP inhibited inflammatory pain triggered by formalin in a dose-dependent manner [5]. They indicated that formalin-triggered inflammatory pain was associated with the initiation of peripheral and spinal mitogen-activated protein kinases (MAPKs) both in Phase I and II [5]. Nevertheless, the above reaction was blocked by pretreatment with AGAP. Thus, they concluded that pretreatment with AGAP blocked the spinal Fos secretion triggered by formalin. Their study also revealed that AGAP potentiated the consequences of the inhibitors of MAPKs on inflammatory pain (Figure 1) [5].

Ruan et al. demonstrated that intrathecal administration of AGAP both inhibited and reversed chronic constrictive injury (CCI)-triggered by thermal hyperalgesia and mechanical allodynia [1]. They further indicated that triggering CCI augmented secretion of p-MAPKs and spinal Fos which was also inhibited or reversed by treated AGAP. Furthermore, AGAP relieved pain linked with formalin-triggered inflammation and regulated formalin-related augmented secretion of p-MAPK and spinal Fos (Figure 1). Thus, they concluded that AGAP potentiates the effects of MAPK inhibitors on neuropathic and inflammation-associated pain [1].

## 5. Mechanisms via Which BmK AGAP Elicits Analgesia

Liu et al. indicated that intrathecal administration of AGAP in a dose-determined manner reduced formalin-triggered spontaneous nociceptive behaviors as well as spinal c-Fos secretion in rats [40]. Ma et al. demonstrated that rAGAP and its 12 mutants were notably dissimilar from the negative control with respect to analgesic action [10]. They explained that the region responsible for analgesic action was numerous and mutants in disulfide bridges with partially damaged sections still have a native-like structure. Nevertheless, they found out that the analgesic actions of the 12 mutants were lower than those of rAGAP, which suggested that the existence of all disulfide bonds appeared to be essential for the analgesic action of the rAGAP [10].

Also, two mutants in Cys22S, Cys46S, and Cys16S, Cys36S had relatively lower actions, which means that the region of analgesic action was obviously disrupted [10]. Furthermore, the disulfide bond Cys16-Cys36 was associated with the long-loop of the *β*-strand II, while the bridge Cys22-Cys46 was associated with the *α*-helix of the *β*-strand II. The *β*-strand II was linked to the *β*-strand III to form a *βαβ* motif comprising six key-chain hydrogen bonds [10, 41]. Ma et al. indicated that, in Cys16-Cys36 and Cys22-Cys46, the disulfide bonds were interrupted and the six key-chain hydrogen bonds also fragmented leading to augmented flexibility of the long-loop as well as *β*-turn II [10].

Mutants in Cys26S and Cys22S had a substantial effect on the analgesic action of AGAP. Also, amino acids Cys22 and Cys26 were detected in the *α*-helix which was associated with the *β*-strand III to form the *α*/*β* skeleton [42]. This framework was well-maintained in all the long-chain scorpion toxins described in the literature [42]. Furthermore, in mutants Cys22S and Cys26S, the entire structure may generate a major change, mainly in the analgesic region leading to a decrease in analgesic action [42].

On the other hand, Cys46S preserved 99.46% mutational action relatively, which indicated that the domain of analgesic action was practically unbroken [10]. Moreover, it was observed that, though the *β*-strand content of Cys36S generated a relatively reduced analgesic activity, it preserved 90.36% relative analgesic action. Also, Cys36 sited in the *β*-strand II may contribute to structural stability [10]. The analgesic action of its replacement did not reduce tersely, which means that the disulfide bonds played a crucial role in its regulatory activity [10, 43]. Kampo et al. demonstrated that AGAP had an analgesic activity which may be an effective therapeutic agent for pain management because of its downregulation of PTX3 via NF-*κ*B and Wnt/*β*-catenin signaling pathway (Figure 1) [44]. Further studies are required in this direction.

## 6. Signing Pathways via Which BmK AGAP Function

Studies have proven that modulation of MAPKs contributed to distinctive nociceptive activities and peripheral central sensitization triggered by distinctive noxious stimuli [45–48]. MAPKs, comprising p38, extracellular signal-regulated protein kinase (ERK), and Jun N-terminal kinase (JNK), are a family of serine/threonine protein kinases that transduce extracellular stimuli into intracellular posttranslational and transcriptional reactions (Figure 1) [46, 47]. Ruan et al. found that MAPKs signaling facilitated the function of AGAP via inhibiting neuropathic and inflammation-associated pain [1]. Also, AGAP potentiated the actions of MAPK inhibitors in regulating inflammation-associated pain (Figure 1) [1].

AGAP was capable of downregulating the secretion of phosphorylated p38 (p-p38), phosphorylated JNK (p-JNK), and phosphorylated ERK 1/2 *in vitro* (Figure 1) [7]. Ruan et al. demonstrated that intraplantar administration of AGAP enhanced formalin-triggered impulsive nociceptive behavior, followed by reduced secretion of peripheral as well as spinal phosphorylated (p)-MAPKs [1]. Therefore, it was likely that MAPKs, downregulatory effectors, contributed to the modifying of spinal nociceptive activities associated with AGAP [1]. Nevertheless, spinal ERK signaling was triggered via phosphorylation and phosphorylated ERK which happens to be a marker of pain behavior-related neuronal sensitization [1, 49].

Fos protein secretory levels have been used as markers for neuronal stimulation in the central nervous system [5, 50]. It has extensively been used as a marker for the practical mapping of neuronal circuits in reaction to numerous described stimuli [50, 51]. There was a positive correlation between the amount of Fos protein secretion and the grade of sensitization triggered by nociceptive stimuli in spinal cord neurons [5]. Studies have shown that prolong triggering of the c-fiber-evoked firing of broad active collection neurons in the spinal dorsal horn in reaction to taming stimulation of afferent fibers was accompanied by the frequency-dependent upsurge of c-fos-labeled cells in superficial, intermediate, and a deep laminate of the dorsal horn on the stimulated end [5, 50, 51]. Also, AGAP was capable of inhibiting formalin-induced spinal c-Fos secretion (Figure 1) [1, 5]. Mao et al. affirmed further that preintraplantar administration of AGAP inhibited the secretion of spinal Fos protein in the formalin pain model (Figure 1) [5]. The reduced secretion of spinal Fos protein affirmed the antinociceptive function of pretreatment with AGAP [5].

**Figure 2:**
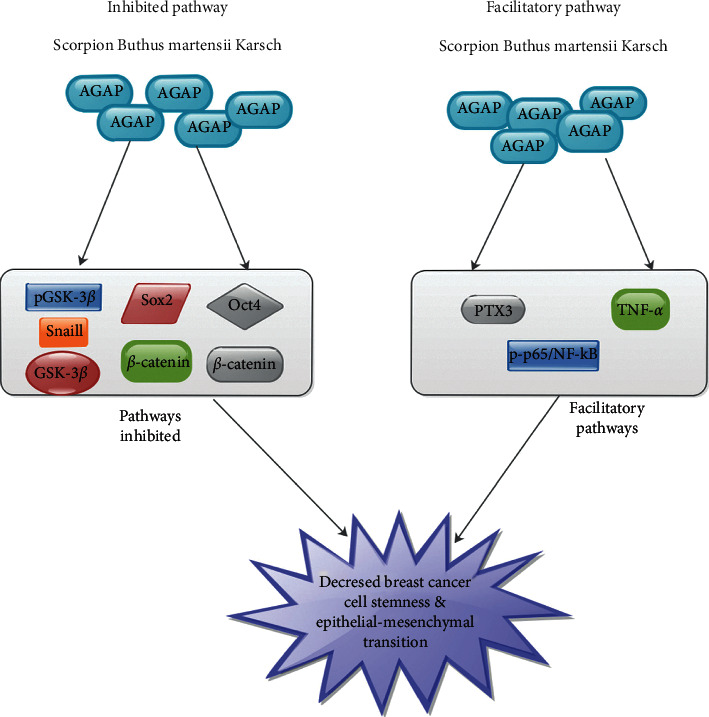
An illustration showing the inhibitory and facilitatory pathways via which AGAP is decreasing breast cancer cell stemness and epithelial-mesenchymal transition.

Studies have shown that *β*-catenin was very crucial in mediating the cross-regulation of NF-*κ*B via the GSK- 3*β* pathway [52, 53]. Kampo et al. discovered that rAGAP blocked pGSK-3*β*, GSK-3*β*, and *β*-catenin *in vitro* and *in vivo* (Figure 2). They indicated that AGAP was capable of reducing the secretion of p-p65/NF-*κ*B, TNF-*α*, and PTX3 in breast cancer cells (Figure 30 in [44]). They thus concluded that AGAP mediated the downregulation of PTX3 via the NF-*κ*B signaling pathway. They further observed that reduced secretion of *β*-catenin, Oct4, Sox2, and Snail1 and augmentation in the secretion of E-cadherin by AGAP both *in vitro* and *in vivo* were capable of decreasing breast cancer cell stemness and epithelial-mesenchymal transition (Figure 2) [44]. Further signing studies are needed to establish a link between AGAP and morphine and other pain receptors.

## 7. BmK AGAP and Apoptosis

Studies have demonstrated that p27 and PTEN/PI3K/Akt pathways are crucial during cell cycle succession. On the other hand, Bcl-2 family proteins modulate mitochondrial membrane permeability and mitochondrial apoptosis pathway [6, 54]. The Bcl-2 family proteins comprise antiapoptotic protein like Bcl-xl, Bcl-2, KSHV-Bcl-2, and Bcl-w and proapoptotic proteins like Bax, Bad, and Bid [55]. Gu et al. demonstrated that rAGAP was capable of influencing apoptosis. They indicated that the secretion of cell apoptosis associated proteins like Bcl-2, Bax, and PTEN/PI3K/Akt pathway was crucial in the molecular mechanism of apoptosis stimulation [6].

Also, AGAP was capable of triggering downregulation of Bcl-2, upregulation of caspase-3, and reduced cell cycle associated protein cyclin D in MCF-7 cells and human lymphoma cells [56]. Gu et al. demonstrated that rAGAP upregulated the secretion of Bax and triggered the concurrent downregulation of Bcl-2, thus reducing the Bcl-2/Bax proportion. Moreover, the PTEN/PI3K/Akt pathway was also implicated in cell apoptosis succession [6]. They indicated that rAGAP appreciably augmented secretion of PTEN and reduced secretion of PI3K and phosphorylation stimulation of Akt [6].

## 8. BmK AGAP and Cell Cycle

Cell cycle comprises the pre-DNA synthesis phase G1, DNA synthesis phase (S), DNA postsynthetic phase G2, and the phase of mitosis M. It was proven that cells are capable of halting mitosis and moving from the G1 phase of the cell cycle into G0 phase which is usually a temporary stationary phase [57]. Cell cycle arrest is the crucial mechanisms via which antitumor medication elicits the function. Also, if the cell cycle is inhibited at the G1 phase, the unlimited proliferation of tumor cells would be modulated [58]. Gu et al. demonstrated that rAGAP was capable of inhibiting the adaptability of SW480 cells. They further indicated that rAGAP triggers SW480 cell cycle arrest at the G0/G1 phase, followed by a decrease in the S phase but no substantial modification in the G2/M phase [6]. Thus, the cell cycle did attain the G1/S restriction phase and was blocked from going through the G1 to S phase [59, 60].

p27, a member of the CDK blocker family, is a tumor suppressor gene that inhibits phosphorylation of the Rb protein and accordingly blocks cell growth and proliferation [6]. Studies have shown that the PI3K pathway played a critical role during cell cycle progression. This pathway was triggered in the G1/S transition leading to cell proliferation, growth, and resistance to cancer therapy [61, 62]. Furthermore, PTEN was a tumor suppressor gene regulated via the PI3K/Akt pathway [63]. Studies have proven that p-Akt phosphorylates proteins like NF*κ*B, mTOR, Bad, GSK-3*β*, and MDM-2 leading to the augmentation of cell growth, metabolism, survival, and proliferation once it was activated [64, 65]. Moreover, the PTEN/PI3K/Akt pathway blocked G1/S cell cycle progression and G2/M transition resulting in defects in DNA damage checkpoint control when it was constitutively activated [66].

Gu et al. demonstrated that rAGAP upregulated the secretion of p27 and PTEN, while it downregulated PI3K and p-Akt secretion. They established that rAGAP was capable of increasing the quantity of p27 protein and blockade PI3K/Akt signal transduction resulting in G0/G1 cell cycle arrest in SW480 cells [6]. Zhao demonstrated that the cell cycle was triggered by rAGAP in SHG-44 cell arrested in the G1 phase, followed by a decrease in the S phase with no alteration in the G2/M phase [7]. They further indicated that the cell cycle from G1 to S phase was modulated via cyclin D/CDK4 (CDK6) or cyclin E/CDK2 multiplexes. Cyclin/CDK multiplexes intermediated RB phosphorylation in the late G1 phase, and hyperphosphorylated RB triggered the secretion of transcription factor E2F, leading to cell cycle progression to S phase [7]. Also, the downregulation of p-AKT resulted in a decreased secretion of CDK2 and CDK6 leading to a decrease in the protein levels of p-RB. Thus, this pathway was capable of influencing cell cycle arrest in the G1 phase and blockade of the proliferation of SHG-44 cells [7].

## 9. BmK AGAP and Cancer

**Figure 3:**
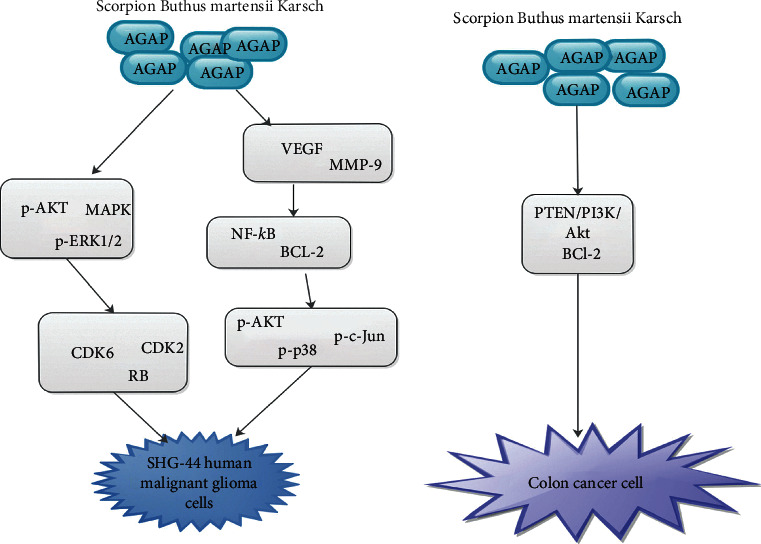
An illustration showing the pathways via which AGAP influence SHG-44 Human Malignant Glioma Cells and colon cancer cell.

Liu et al. indicated that AGAP has an antitumor effect against *E. ascites* tumor and S-180 fibrosarcoma. They further indicated that AGAP was more effective for tumor cells and less harmful for healthy cells than cyclophosphamide [4]. Additionally, rAGAP was capable of blocking the proliferation of lymphoma and glioma [3]. Thus, rAGAP is a promising antitumor therapy [3]. Hence, further studies are needed in this direction. Also, comparative studies between the antitumor potentials and already established anticancer medications are warranted. Gu et al. indicated that the intrinsic apoptotic pathway modulating the Bcl-2 family and the PTEN/PI3K/Akt pathway was associated with rAGAP-triggered SW480 colon cancer cell (CRC) apoptosis (Figure 3). Thus, rAGAP was a potential therapeutic agent for CRC [6].

Zhao et al. demonstrated that AGAP downregulated the protein secretion of p-AKT and p-Erk1/2 resulting in a decrease in the protein concentrations of CDK2, CDK6, and p-RB, leading to G1 cell cycle arrest (Figure 3) [7]. These alterations resulted in the blockade of SHG-44 cells proliferation. They further indicated that AGAP was capable of blocking the proliferation and migration of SHG-44 cells leading to suppressing of protein secretory levels of VEGF and MMP-9 via downregulation of NF-kB and BCL-2 which were modulated via the triggering of p-AKT, p-p38, and p-c-Jun (Figure 3) [7]. They concluded that, when the concentrations of rAGAP were elevated, the protein levels of NF-kB and BCL-2 in treated SHG-44 cells were gradually downregulated via suppressing the protein secretory levels of p-AKT, p-Erk1/2, p-c-Jun, and p-p38 in a dose-dependent manner [7]. Nevertheless, there was a decrease in the concentration of VEGF and MMP-9 via the modulation of NF-kB and BCL-2. Zhao et al. further revealed that the migration of SHG-44 cells in a wound healing assay was associated with Erk, p38, c-Jun, MAPK, and AKT pathway (Figure 3). Thus, rAGAP was capable of blocking the proliferation of SHG-44 cells via suppressing the modulation of p-Erk1/2. They stressed in their experiments involving the DNA ladder that rAGAP induced SHG-44 cells in a dose- and time-dependent manner, but the DNA ladder did not occur [7].

PTX3 was capable of triggering inflammation activities as well as complement [67]. Also, PTX3 was capable of recruiting leukocyte into inflamed tissues and clearance of apoptotic cells [68]. Bonavita et al. demonstrated that PTX3 influences tumor associated inflammation and chemoresistance during breast cancer treatment [69]. Basile et al. established that NF- *κ*B binding site, p65/NF-*κ*B was functionally significant in PTX3 promoter such as TNF-*α*. They indicated that the activities of NF-*κ*B in TNF-*α* were essential for the molecular mechanisms vital for the modulation of PTX3 [70]. Nevertheless, the secretion of PTX3 in breast cancer was elevated and linked to stem-like structures, epithelial-mesenchymal transition, migration, invasion, and metastasis [71]. Kampo et al. demonstrated that AGAP blocked cancer stemness and epithelial-mesenchymal transition via downregulating PTX3 secretion in breast cancer [44]. They indicated that AGAP intermediated downregulation of PTX3 via NF-*κ*B and Wnt/beta-catenin signaling pathways. They concluded that AGAP had analgesic activity which may be an effective therapeutic agent for cancer [44].

## 10. Conclusion

Our review points clearly to the fact that AGAP has analgesic and antitumor potentials. AGAP had a sturdy inhibitory influence on both viscera and soma pain. VGSCs are present in excitable membranes and partake in essential roles in action potentials generation as compared to the significant function of VGCCs. AGAP potentiates the effects of MAPK inhibitors on neuropathic and inflammation-associated pain. AGAP has more affinity for tumor cells and has less harmful effects on healthy cells. In cancers like colon cancer, breast cancer, lymphoma, and glioma, rAGAP was capable of blocking proliferation. Thus, AGAP is a promising analgesic and antitumor therapy for these tumors. Nevertheless, research is needed with other tumors.

## Data Availability

No data were used to support this paper.
